# Thermophoresis and thermal orientation of Janus nanoparticles in thermal fields

**DOI:** 10.1140/epje/s10189-022-00212-3

**Published:** 2022-07-09

**Authors:** Fernando Bresme, Juan D. Olarte-Plata, Aidan Chapman, Pablo Albella, Calum Green

**Affiliations:** 1 Department of Chemistry, Molecular Sciences Research Hub, Imperial College London, London, W12 0BZ UK; 2 Department of Applied Physics (Group of Optics), University of Cantabria, Avda. Los Castros, s/n, Santander, 39005 Spain

## Abstract

**Abstract:**

Thermal fields provide a route to control the motion of nanoparticles and molecules and potentially modify the behaviour of soft matter systems. Janus nanoparticles have emerged as versatile building blocks for the self-assembly of materials with novel properties. Here we investigate using non-equilibrium molecular dynamics simulations the behaviour of coarse-grained models of Janus nanoparticles under thermal fields. We examine the role of the heterogeneous structure of the particle on the Soret coefficient and thermal orientation by studying particles with different internal structures, mass distribution, and particle–solvent interactions. We also examine the thermophoretic response with temperature, targeting liquid and supercritical states and near-critical conditions. We find evidence for a significant enhancement of the Soret coefficient near the critical point, leading to the complete alignment of a Janus particle in the thermal field. This behaviour can be modelled and rationalized using a theory that describes the thermal orientation with the nanoparticle Soret coefficient, the mass and interaction anisotropy of the Janus nanoparticle, and the thermal field’s strength. Our simulations show that the mass anisotropy plays a crucial role in driving the thermal orientation of the Janus nanoparticles.

**Graphic abstract:**

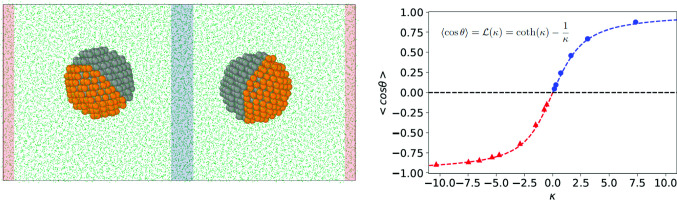

**Supplementary Information:**

The online version contains supplementary material available at 10.1140/epje/s10189-022-00212-3.

## Introduction

Thermal gradients induce thermophoretic forces on colloidal suspensions. In aqueous suspensions, the colloids migrate towards cold (thermophobic) or hot regions (thermophilic) depending on the average temperature and chemical composition of the suspension [1–5]. This behaviour follows the observations of Ludwig and Soret [6, 7] using alkali halide aqueous solutions, where the thermal gradients lead to concentration gradients. The magnitude of this mass/heat flux coupling effect is often quantified using the Ludwig-Soret coefficient (or Soret coefficient).

The Soret coefficient of colloids depends on several variables. The colloidal mass, size, and charge are amongst the most widely investigated thus far, see e.g. references [5, 8–12] for systematic analyses of some of these variables using atomistic, mesoscopic simulations, theory and experiments. The coupling of internal degrees of freedom of molecules, colloids with heat fluxes leads to molecular, particle orientation [13, 14]. If the colloids are anisotropic, e.g. rod like shape, this leads to distinctive changes in the diffusion coefficient of the colloids and simultaneous changes of the Soret coefficient, which feature a maximum with increasing mass, instead of the monotonic increase of the Soret coefficient observed in spherical colloids [15].

Janus nanoparticles (JNPs) [16, 17] are heterogeneous colloids consisting of two components, which can have significantly different properties, e.g. hydrophilicity and molecular mass. The heterogeneous structure of the nanoparticle results in fairly complex phase diagrams [18, 19], and these particles have attracted interest in the area of active matter, particularly self-thermophoretic motility [20]. Self-thermophoresis was achieved by coating half of a colloid with a metallic layer that can be heated using light. The light–matter interaction leads to local heating and a thermal gradient around the Janus particle and self-propulsion.

The intrinsic anisotropy of JNPs, both solvent–particle interactions and internal mass distribution, drives the thermophoresis and thermal orientation of the particles. Generally, strong solvent interactions (e.g. hydrophilic) induce nanoparticle motion towards cold regions, *i.e.* thermophobicity. Similarly, Janus particles consisting of two different materials will experience a torque, and the region with the stronger interactions with the solvent will orient towards cold regions [21, 22]. However, JNPs do also feature mass anisotropy. When the mass ratio of the two Janus components is large enough, the orientation can be reversed, with the stronger interactions facing the hot region [22]. The thermal orientation effect emerges from the intrinsic internal anisotropy of the JNPs, and it is of particular interest in the context of recent experiments of Janus colloids, where “polarization” (i.e. orientation) was reported in the presence of thermal fields [23].

In this work, we investigate the thermophoretic force and thermal orientation of JNPs as a function of the particle’s mass, interaction anisotropy and fluid temperature and density. We use coarse-grained models of uncharged JNPs with different internal compositions: two hemispheres with different materials, or JNPs with a thin layer in one of the hemispheres. The latter models mimic the anisotropic structures used in active matter to study “polarization” effects in thermal gradients. We will show that mass anisotropy plays a crucial role in defining the orientation of the nanoparticles. On the other hand, the interaction strength plays a relatively minor role in the heavy nanoparticles studied here.

## Models and methods

We performed simulations using a coarse-grained model. The solvent and the nanoparticle are represented with Lennard-Jones particles that interact according to the potential,

1 $$\begin{aligned} u_{ij}(r)= \left\{ 4 \varepsilon _{ij} \left[ \left( \frac{\sigma }{r} \right) ^{12} - \left( \frac{\sigma }{r} \right) ^6 \right] - u(r_c) \right\} \theta (r_c-r)\nonumber \\ \end{aligned}$$

where $\varepsilon _{ij}$ is the interaction strength between particles of species $i,\, j$, $\sigma$ is the diameter of the particles in the solvent and in the JNP, *r* is the interaction distance between particles $i,\, j$, $\theta (r)$ is the Heaviside step function and $r_c =2.5\sigma$ is the interaction cut-off. We use hereafter reduced units for the temperature, $T^* = k_B T/\varepsilon _\mathrm{solvent}$ and density, $\rho ^* = \rho \sigma ^3$, defined in terms of the interactions strength, $\varepsilon _\mathrm{solvent}$, and diameter, $\sigma$, of the solvent.

**Fig. 1:**
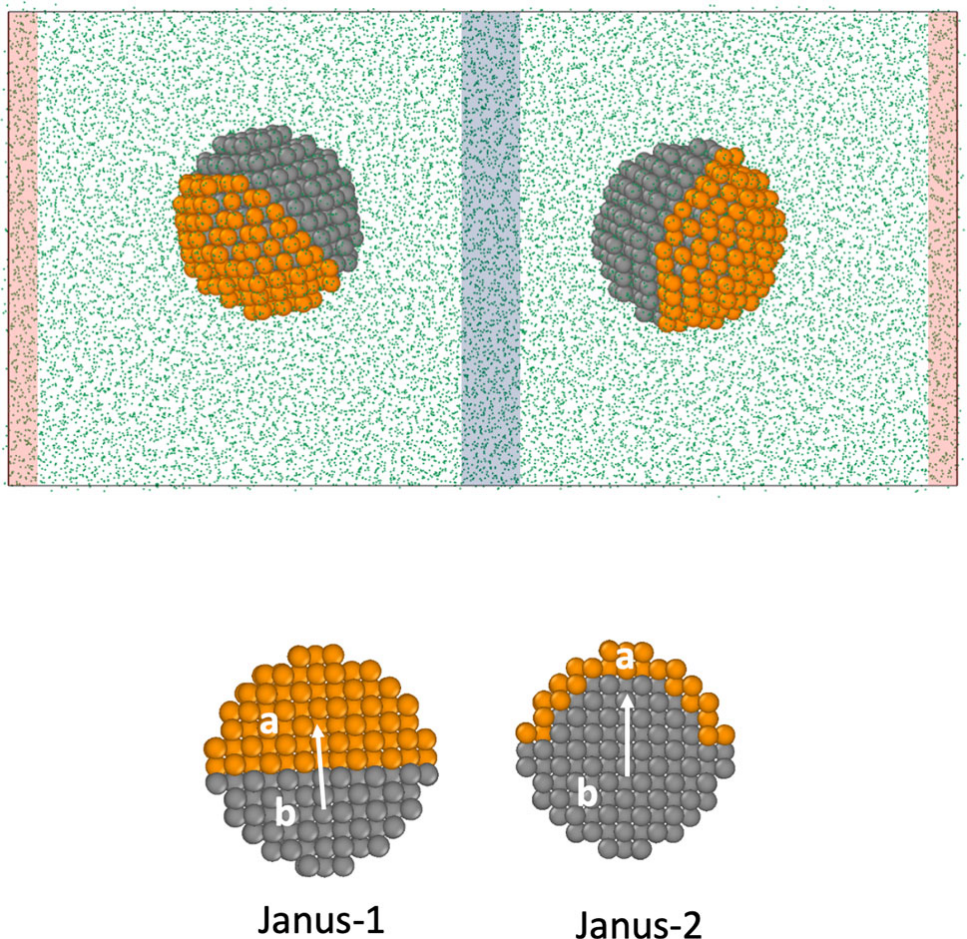
(Top) Snapshot of the simulation box employed in this work. The red (hot) and blue (cold) shadowed areas indicate the location of the thermostatting regions. The JNPs are represented in grey and orange, and the solvent as green dots. (Bottom) A cross-sectional view of the two types of JNPs investigated in this work. The arrows represent the unit vector defining the orientation of the JNPs, $\mathbf{u}_c$ in the main text. “a” and “b” indicate the two different hemispheres of the “Janus 1” nanoparticle or the shell and core parts of the “Janus 2” nanoparticle. Simulation snapshots were visualized using OVITO [24] .

**Fig. 2:**
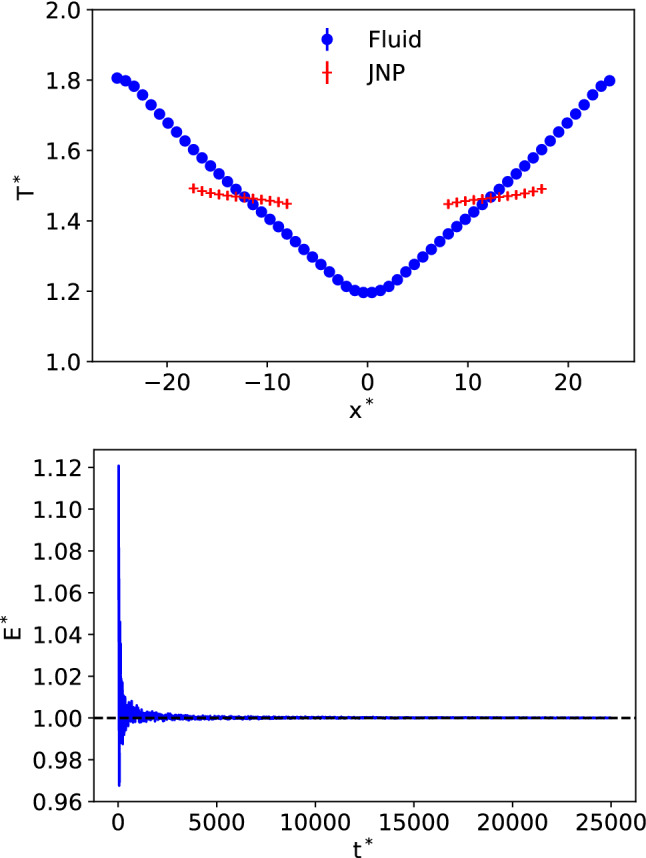
(Top) Representative temperature profiles of the fluid (blue) and colloids (red). The fluid temperature was calculated using all the particles in the simulation box. (Bottom) Ratio of cumulative energy exchanged at cold and hot thermostatting regions, $E^*=| E_\mathrm{cold}/E_\mathrm{hot} |$ as a function of time. “1” indicates the energy exchange is identical.

We performed Non-Equilibrium Molecular Dynamics simulations using a cuboid box (see Fig. 1). The velocities of the particles in the thermostatting regions (see red-hot and blue-cold in Fig. 1) were reset every 100 timesteps using a simple velocity rescale. The particles outside the thermostatting regions, including the nanoparticles, followed Newtonian dynamics. The application of the thermostats generates well-defined temperature gradients for the fluid and the nanoparticles (see Fig. 2-top) and heat fluxes obtained from the energy exchange rate at the thermostats (see Fig. 2-bottom). The amount of energy exchange (following a short transition period) is the same in the cold and hot thermostats, showing that our method provides excellent energy conservation. The temperature difference between cold and hot thermostats was set to $T_\mathrm{HOT}^* = T_\mathrm{COLD}^* + 0.6$. The temperature and orientation profiles were computed by dividing the simulation box into layers of thickness 0.84$\sigma$ in the thermal gradient direction (*x*).

We used a crystalline fcc lattice, with density $\rho ^*=(N/V) \sigma ^3 = 1$, to construct the Janus nanoparticles. We built two types of Janus nanoparticles: a Janus nanoparticle (Janus-2) consisting of a thin “shell” of particles at the surface of the particle coating half of the “core” particles, and a Janus nanoparticle (Janus-1) consisting of two hemispheres with particles of different types. Hereafter, we will refer to the two types of particles in the JNP as “a” and “b”. We show in Fig. 1-bottom cross-sectional views of both types of nanoparticles. The nanoparticles had an average radius of $R \sim 5 \sigma$. The interactions between all the particles inside the nanoparticle irrespective of the particle type were set to $\varepsilon _\mathrm{Janus}/\varepsilon _\mathrm{solvent}=20$. The Janus-2 nanoparticles contained $n_a=$129 shell particles and $n_b=$402 core particles, and the Janus-1 nanoparticles, $n_a=$296 and $n_b=$235. For the analysis presented below, we varied the interaction strength and/or mass of the particles of type “a” in both Janus-1 and Janus-2 nanoparticles (see Fig. 1).

We computed the Soret coefficient and orientation of the nanoparticles as a function of the mass ratio, $M_a/M_b=n_a m_a/(n_b m_b)$, total mass of the nanoparticle, and solvent interaction strength ratio $\varepsilon _{a}/\varepsilon _{b}$. The Soret coefficient was calculated from the average force acting on each nanoparticle in the direction of the heat flux [8, 10],

2 $$\begin{aligned} S_T = \frac{f_\mathrm{solvent} - f_\mathrm{Janus}}{k_B T \nabla T} \end{aligned}$$

where $f_\mathrm{solvent}$ is the thermophoretic force on the solvent (in the direction of the heat flux) and it is of the order of $f_s \sim k_B \nabla T$ (see reference [14] for a test of this relationship in a solvent similar to the one employed here). The Soret coefficient is $S_T >0$, for thermophobic particles, *i.e.* particles migrating preferentially towards the cold region. The displacement of the colloids was restrained using a harmonic potential, $U_r^* = \frac{k^*}{2} (r^*-r_e^*)^2$, where $k^*=1000$ is the force constant, $r^*$ represents the center of mass coordinates of the nanoparticle and $r_e^*$ is the initial position of the nanoparticles in the simulation box ($r^*_e= L_x/4$ and $r^*_e= 3 L_x/4$). We calculate in the simulations the average displacement of the nanoparticles with respect to $r^*_e$. The product of the displacement times the force constant gives the thermophoretic force.

The orientation of the Janus nanoparticles with respect to the direction of the heat flux is defined by $\langle \cos \theta \rangle = \mathbf{u}_\mathrm{Janus} \cdot \mathbf{u}_{\mathbf{J}_{q}}$ where, $\mathbf{u}_\mathrm{Janus}$ and $\mathbf{u}_{\mathbf{J}_{q}}$ are the unit vectors for the Janus colloid (see Fig. 1-bottom) and the heat flux, respectively. The average orientation is defined as $\langle \cos \theta \rangle <0$ when the particles of type “a” in the nanoparticles orient in the direction of the hot thermostat.

To analyse the local structure of the solvent around the nanoparticles, we calculated the radial density profiles using *homogeneous* nanoparticles with nanoparticle–solvent interaction set to $\varepsilon _{a} = \varepsilon _b$. By setting the same interaction, we obtain angle-independent radial density profiles and hence increase the statistics by using all the fluid particles surrounding the spherical particle. Solvent–solvent interactions were set to $\varepsilon _\mathrm{solvent}=1$. The simulations were performed at equilibrium (NVT) conditions. The NEMD set-up for the core-shell particles was used for simplicity, with both thermostat temperatures set to the same temperature. Six representative values of $\varepsilon$ were used to compute the density profiles at three different temperatures ($T^*$ = 1.1, 1.5, 2.5) and for two different fluid densities, $\rho ^*$ = 0.4, 0.8, targeting relevant thermodynamic states (see Fig. 4). Spherical binning was performed around both nanoparticles. The profiles around each nanoparticle were averaged over 5 statistically independent replicas. The error bars represent the standard error from independent repeats.

We also computed the radial density profiles around non-Janus core-shell nanoparticles, with the shell coating the entire nanoparticle. These particles had $n_a = 282$ and $n_b = 249$ shell and core atoms, respectively. The thickness of the shell was the same as in the Janus-2 nanoparticle. Interaction strengths $\varepsilon _a/\varepsilon _b = 10,\ 20$ were considered to study the fluid structure in the high particle–fluid interaction regime. These simulations were performed at the same temperatures and fluid densities indicated above.

The NEMD simulations involved 30 independent simulations, spanning 10$^7$ steps each. These trajectories were used to obtain statistical averages and statistical errors (standard error). The simulation trajectories were integrated using LAMMPS [25, 26] and a time step of $\delta t^* = 0.0025$.

Typical simulation boxes with small systems consisted of 13,117 (low density) or 26,401 (high density) particles. We performed additional computations with different systems sizes, and number of solvent particles varying in the interval 10$^4$-2$\times 10^5$. The simulations were performed in cuboid boxes with dimensions $(L^*_x, L^*_y,L^*_z) = (50.796,25.398,25.398)$ for the small systems, and $(L^*_x, L^*_y,L^*_z) = (101.594,50.796,50.796)$ for the largest system.

## Results

### Simulation conditions

**Fig. 3:**
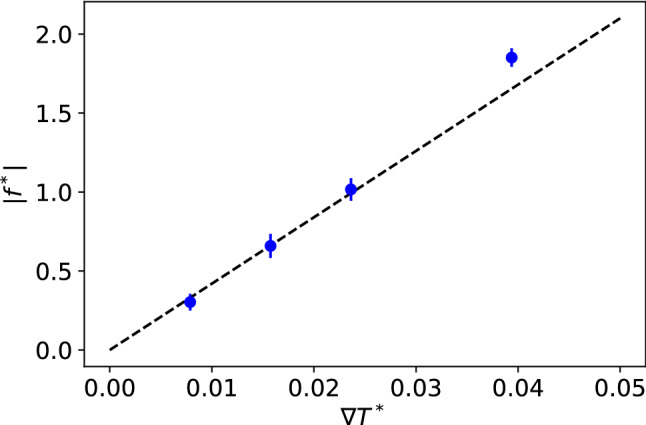
Dependence of the absolute value of the thermophoretic force of the nanoparticle, $f_\mathrm{Janus}$, with the magnitude of the thermal gradient. The average temperatures of the nanoparticles for the different runs are: (T$^*_\mathrm{HOT}$, T$^*_\mathrm{COLD}$, T$^*_\mathrm{avg}$) = (1.6, 1.4, 1.47), (1.7, 1.3, 1.44), (1.8, 1.2, 1.45), (2, 1, 1.38). All the simulations were performed with the Janus-2 nanoparticles and the following conditions: $\varepsilon _{b} = 10$, $\varepsilon _{a} = 20$, $m_{b} = 1$, $m_{a} = 20$, nanoparticle radius $R^* = 5$.

Before performing production computations, we analysed the dependence of the thermophoretic force with the strength of the thermal gradient. Our results conform to a linear response for $\nabla T^* \le 0.025$ (see Fig. 3). The largest gradient ($\nabla T^* = 0.04$) fulfills the inequality ($2R \nabla T^*/ T^* = 0.17 < 1$), but the temperature of the colloid is $\sim 5$ % lower than that obtained with the smaller gradients (see caption of Fig. 3 for additional information). Hence, we performed the production simulations using a temperature difference $T^*_\mathrm{HOT} - T^*_\mathrm{COLD} = 0.6$, corresponding to $\nabla T^* = 0.025$. We have shown before that the thermal conductivity obtained from NEMD using a large thermal gradients, $\nabla T^* \sim 0.04$, agrees with the results from equilibrium Green-Kubo computations [27]. Hence, the thermal conductivity of the solvent is expected to be independent of the thermal gradient for the thermal gradients employed in our work.

**Fig. 4:**
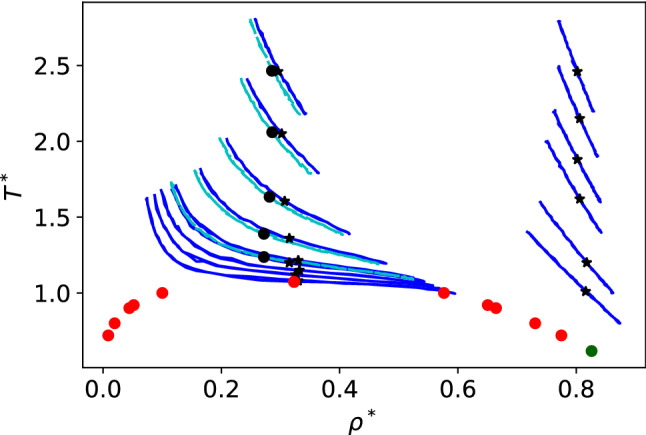
Temperature versus density and phase diagram for the spherically truncated and shifted Lennard-Jones potential (Eq. (1)). The red points are taken from reference [28]. The estimated critical point for this model is $T_c^* = 1.073$, $\rho _c^* = 0.323$. The triple point (green) is taken from reference [29], $T^*_t = 0.617$, $\rho _l = 0.825$. The blue lines represent the equations of state of the systems simulated using non-equilibrium simulations. The cyan lines at low density represent NEMD simulations at pressures slightly different from the closest blue lines. The circles and stars signal the density and temperature conditions for the JNPs in each simulation.

We show in Fig. 4 the isobars corresponding to the thermodynamic states investigated in this work. These isobars were obtained by representing the pairs ($T^*,\, \rho ^*$) generated in the NEMD simulations. It has been demonstrated that the isobars obtained from NEMD in this way match the equilibrium results, hence supporting the local equilibrium hypothesis (see e.g. Ref. [30]). The JNPs were immersed in dense liquid phases, dense supercritical fluids, and low-density supercritical fluids with an average fluid density close to the critical density of the Lennard-Jones solvent investigated here. The isobars in Fig. 4 show that the thermal expansion varies significantly with temperature, decreasing with increasing temperature or pressure (see the slope of the isobars corresponding to high-density fluids). The isobars near the critical point show a significant thermal expansion. As we will show below, the Soret coefficient and thermal orientation of the nanoparticles vary significantly upon approaching the critical point.

Thermoosmotic effects appear at liquid-solid interfaces in the presence of thermal gradients, leading to pressure differences induced by temperature differences. We computed the pressure profile in the direction parallel and perpendicular to the centre of mass of a nanoparticle with homogeneous composition (see Fig. 1 in the SI). To do this we used a cylindrical sampling volume of radius $0.8\sigma$, whose axis passes through the tethering point of the nanoparticle ($r^*_e$), and obtained the fluid pressure profile for regions of the nanoparticle pointing towards the hot or cold thermostats. We do not find noticeable pressure differences between hot/cold regions outside the interfacial fluid-nanoparticle interface. Next to the nanoparticle surface the pressure profile features significant changes due to presence of the nanoparticle-fluid interface.

### Nanoparticle–solvent interactions

**Fig. 5:**
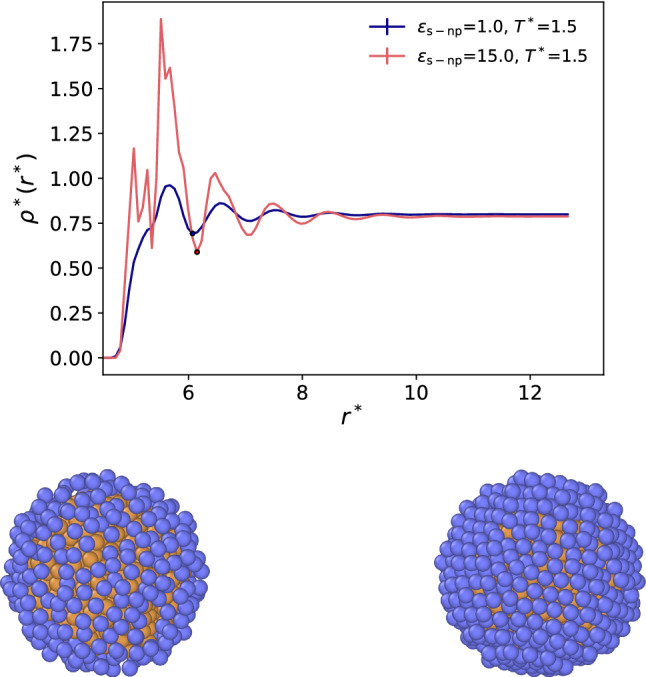
(Top panel) Solvent (s) density profiles around nanoparticles (np) with $\varepsilon _\mathrm{s-np}=1.0$ (black line) and 15 (red line), with $\varepsilon _\mathrm{s}=1$. The simulations were performed at reduced temperature $T^*=1.5$, and solvent bulk density of $\rho ^*=0.8$. (Bottom panels) The simulation snapshots show the solvent particles within a radius up to the first minima in the density profiles from the nanoparticle centre (indicated by the black points in the top figure), corresponding to the nanoparticle first solvation shell. The left panel corresponds to $\varepsilon _\mathrm{s-np}$=1.0 and the right panel to $\varepsilon _\mathrm{s-np}$=15.0. Simulation snapshots were visualized using OVITO [24].

Figure 5-top shows the solvent-nanoparticle (s-np) density profiles for weak and strong solvent–particle interactions. The solvent density reaches the bulk value at $r^*\sim 8\sigma$, much shorter than the smaller box length investigated. Hence, the box size should not influence the solvation structure. We performed additional simulations with bigger simulation boxes (see Fig.2 in the SI) which confirm the lack of dependence of the solvation structure with system size. The profiles show significant changes in the nanoparticle-solvent interfacial structure. Strong interactions ($\varepsilon _\mathrm{s-np} = 15$) lead to a well-defined solvent layer at the nanoparticle surface. The first solvent layer ($r^* < r^*_\mathrm {min}$, the first minimum in the density profile, see Fig. 5-top) fully coats the nanoparticle surface, and it features epitaxial ordering (see snapshot in Fig. 5-bottom). For a weaker interaction strength ($\varepsilon _\mathrm{s-np} = 1$), there is still significant solvent adsorption on the nanoparticle surface. An obvious difference between the weak and strong interactions is the substantial lateral structuring observed in the latter. This is reflected in the surface packing, which is much higher for the stronger interaction strength. By integrating the (number) density profiles up to the first minimum, we compute the average number of atoms in the first solvation shell to be $\sim$524 and $\sim$342 for $\varepsilon _\mathrm{s-np} = 15$ and $\varepsilon _\mathrm{s-np} = 1$, respectively.

**Fig. 6:**
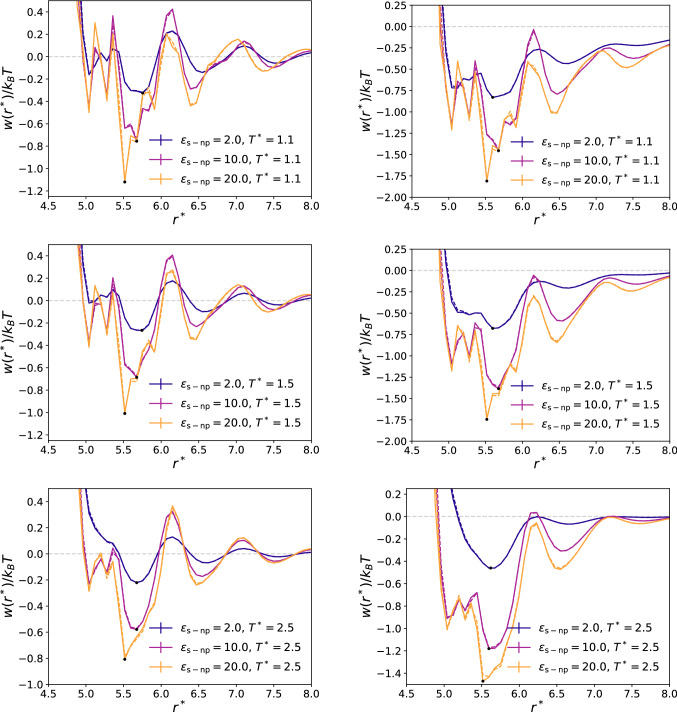
Radial PMF of homogeneous (full lines) and shell-coated (dashed lines) nanoparticles. The left panel corresponds to the high density solvent ($\rho ^*_f\approx 0.8$) and the right panels to the low density solvent ($\rho ^*_f\approx 0.4$). The two labels in each plot represent the solvent–nanoparticle interaction strength and the reduced temperature.

We have quantified the effective solvent–nanoparticle interactions by computing the Potential of Mean Force (PMF),

3 $$\begin{aligned} \frac{w(r^*)}{k_B T}=\frac{w^*(r^*)}{T^*}=- \ln \left[ \frac{\rho ^*(r^*) }{\rho ^*_0 }\right] , \end{aligned}$$

where $\rho ^*(r^*)$ is the density at a distance $r^*$ from the center of geometry of the colloid and $\rho ^*_0$ is the bulk density of the fluid. The bulk density was calculated by averaging the density profiles between $r^*=9$ and the edge of the simulation box. The minima in $w^*(r^*)$ correspond to maxima in $\rho ^*(r^*)$, indicating favourable nanoparticle–solvent interactions. We show in Fig. 6 the PMFs around the nanoparticles at $T^*=1.1, 1.5, 2.5$, interaction strengths, $\varepsilon _\mathrm{s-np}=2, 10, 20$, and high ($\rho _f^*=0.8$) and low ($\rho _f^*=0.4$) solvent densities. These densities correspond to conditions typical of a dense fluid and densities close to the critical one, respectively.

The effective interactions change across different systems, with minima − 0.2$\cdots -1.1$ k$_B$T at high density (see left panels in Fig. 6). Deeper minima are observed in systems with low fluid densities, − 0.4$\cdots -1.8$ k$_B$T (see right panels in Fig. 6). These deeper PMFs for a given $\varepsilon _\mathrm{s-np}$ are connected to the enhancement of the density close to the nanoparticle, relative to the density of the solvent in bulk. The main conclusion from this analysis is that despite the large changes in $\varepsilon _\mathrm{s-np}$ the effective interaction changes, by at most 1.25 $k_B$T units, between $\varepsilon _\mathrm{s-np}=2$ and $\varepsilon _\mathrm{s-np}$ = 10 or 20. The latter interactions are similar to the ones employed in our previous work [22] on JNPs, targeting conditions consistent with those found in JNPs consisting of a polymer core coated with a thin layer of gold. We show in the Supplementary Information (Figure 3-SI) additional results for the general dependence of the first minimum (black dots in Fig. 6) with $\varepsilon _\mathrm{s-np}$ for a wide range of interaction strengths. The smaller dependence of the interaction at $\varepsilon _\mathrm{s-np}$ can be explained by the surface of the particle being saturated with adsorbed solvent atoms. Hence, the effective potential cannot get much stronger with a higher interaction strength, due to the limitation of the number of atoms that can fit in this layer.

The results shown above were obtained with nanoparticles of homogeneous composition. However, the models shown in Fig. 1 and discussed in the next section include Janus-2 nanoparticles featuring a thin coating layer (see Fig. 1-bottom for the Janus-2 nanoparticle). To quantify the impact of the shell on the effective interactions of the nanoparticle, we performed additional simulations using homogeneous nanoparticles coated with a thin layer with the same thickness employed to model the JNPs. The solvent–fluid interaction strength for this layer varied in the interval $\varepsilon _\mathrm{s-np}=\{2\cdots 20\}$, while the interactions for the nanoparticle core was set to $\varepsilon _\mathrm{np}=1$. Our simulations show that the PMFs of shell-coated nanoparticles are equal to the ones obtained with homogeneous nanoparticles. This result shows that the outer-shell interactions dominate the solvation structure, and the underlying weaker interaction of the core does not play a significant role. This observation can be understood considering the interaction cutoff employed here ($r_c=$ 2.5 $\sigma$). For this cutoff, the solvent interacts with two or at most three layers of the nanoparticle. Therefore, the interaction of the outer atoms in the nanoparticle dominates the effective nanoparticle–solvent interactions (see Fig. 6).

The calculations discussed above were obtain using nanoparticles at equilibrium conditions, *i.e.* no thermal gradient. We performed an additional calculation of the solvation structure of the homogenous nanoparticles under a thermal gradient (see Figure 4-SI). The density profiles were obtained using cylindrical bins of radius 0.8$\sigma$ centred at the tethering point ($r^*_e$) of a nanoparticle. Compared to the equilibrium density profiles at a temperature corresponding to the average temperature of the non-equilibrium conditions, the profiles of the thermal gradient show a similar solvent structure at the interface, with a higher or lower ‘bulk’ density depending on whether the profile is measured towards the cold or hot thermostat, respectively. This result shows that the equilibrium simulations reported in Fig. 6 are representative of the local solvation structure around the nanoparticles in the thermal field.

### Soret coefficient and thermal orientation of JNPs at high density

**Fig. 7:**
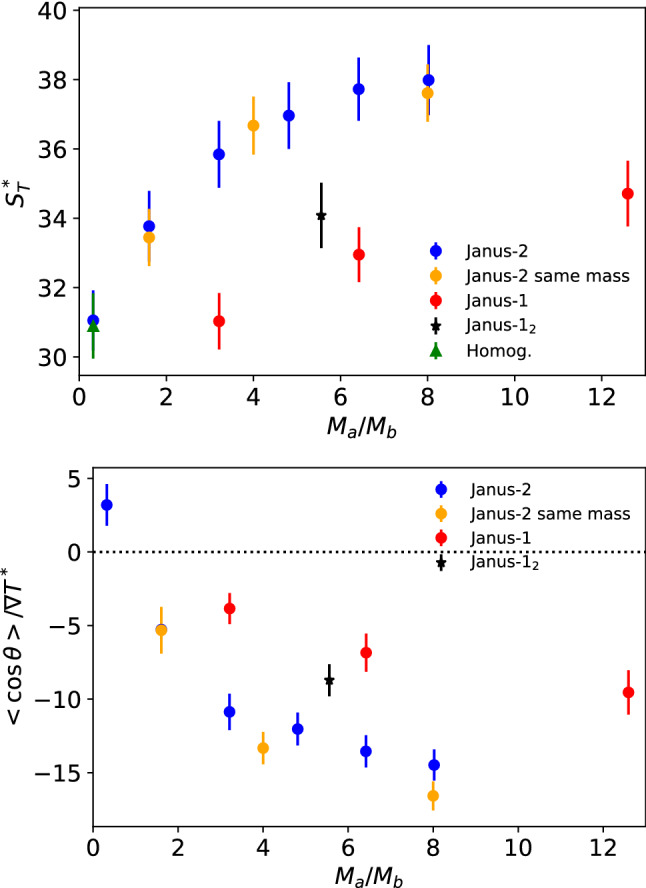
Dependence of the Soret coefficient with the mass ratio, $M_{a}/M_{b}$ for Janus-2 and Janus-1 nanoparticles. The data was obtained using the high density isobar with T$^*_\mathrm{HOT}=$1.8 and T$^*_\mathrm{COLD}=$1.2 (see Fig. 4). The orange symbols represent data at the same thermodynamic conditions, but keeping the overall mass of the nanoparticles constant. The data for the orientation, $\langle \cos \theta \rangle$, has been scaled by the corresponding thermal gradient. All the data was obtained with the interaction strengths: $\varepsilon _{a}=10$, $\varepsilon _{b}=1$, $\varepsilon _\mathrm{solvent}=1$. The masses for the different systems are as follows. $(M_a, M_b)_{\mathrm{Janus-2}}$=(129,402), (645,402), (1290, 402), (1935, 402), (2580, 402), (3225, 402), $(M_a, M_b)_{\mathrm{Janus-2} \, same\, mass}$=(645, 402), (837.6, 209.4), (930.92460, 116.0754), $(M_a, M_b)_{\mathrm{Janus-1}}=(1290.56, 401.85), (2581.12, 401.85), (2960,235)$. The data corresponding to “Janus-1$_2$” correspond to $(M_a, M_b)_{\mathrm{Janus-1_2}}=(1645, 296)$. The data for the “Homog.” nanoparticle were obtained with the Janus-2 nanoparticle by using homogeneous interactions, $\varepsilon _a=\varepsilon _b=1$ and homogeneous mass, $m_a=m_b=1.97175$ for the sites of type a and b. The total mass of this particles is equal to the mass of the “same mass” case, *i.e.* 1047. The orientation for the “Homog.” nanoparticle is $\langle \cos \theta \rangle =0$.

We performed simulations of Janus-2 nanoparticles with a fixed interaction strength ratio $\varepsilon _a / \varepsilon _b = 10/1$, which is of the order of the interaction ratios explored before to model polystyrene-gold-coated JNPs [22]. The interaction $\varepsilon _a=10$, leads to the build up of a strong solvation layer around the nanoparticle shell region (a). The structure of the Janus-2 nanoparticle investigated here mimics more closely the structure of the experimental systems, which consist of a homogeneous core and a coating covering half of the nanoparticle (see Fig. 1-bottom). Figure 7 shows the variation of the Soret coefficient with the mass ratio $M_\mathrm{shell}/M_\mathrm{core} = (n_a m_a)/(n_b m_b)$. The mass of the core was maintained fixed $M_{b}=m_{b} n_{b}=402$ using $m_{b}=1$ and $n_{b}=402$, while the mass of the shell was modified systematically, $M_{a}=m_{a} n_{a}$ with $n_{a}=129$ and $m_{a}=1 \cdots 25$. All the nanoparticles are thermophobic ($S_T > 0$), and therefore have a preference to migrate towards the cold region. The magnitude of the thermophoretic force is significant, taking $\varepsilon _\mathrm{solvent}/k_B =119.3$ K we get $S_T=$ 0.25–0.33 K$^{-1}$ or using the temperature of the solvent at the colloid surface, $T^*=1.5$, we get a thermal diffusion factor $\alpha _T = S_T T \sim 45-60$.

Our Soret coefficients increase with the mass ratio, $M_{a}/M_{b}$. Mass effects have been studied before, and it is known that mass enhances the thermophoretic force [14]. In the interval $M_{a}/M_{b} = 0.3 \cdots 8.0$, the mass of the colloid increases by $\sim 20\%$. However, it is known that mass effects saturate quickly for heavy nanoparticles following the relation $S_T \sim (m_\mathrm{np} - m_s)/(m_\mathrm{np} + m_s)$, where “*np*” and “*s*” refer to the nanoparticle and solvent, respectively [14]. We have analysed the impact of the nanoparticle mass on the Soret coefficient by performing additional simulations of Janus-2 nanoparticles, but changing the mass ratio and keeping the total nanoparticle mass constant. We set the total mass to $M_{T}=M_a + M_b = 1047$, corresponding to an intermediate mass for the Janus-2 particle (see caption of Fig. 7 for numerical values). The Soret coefficients at constant mass agree with the previous coefficients, indicating that mass effects are not driving the observed Soret increase. The independence of the Soret coefficient with the mass of the nanoparticles is consistent with earlier observations that reported a small dependence of the thermal diffusion coefficient and the single-nanoparticle mass diffusion with particle mass [8].

We performed additional simulations with the Janus-1 model (see Fig. 1-bottom-left), sampling total masses in the range $M_{T}= M_a + M_b= 1531 \cdots 2555$, a high mass regime where the Soret coefficient features a weaker dependence with mass ratio. The Soret coefficient of the symmetric JNP does also increase with the mass ratio $M_{a}/M_{b}$, although the Soret coefficients and orientations are noticeably smaller than those obtained with the Janus-2 nanoparticle (see Fig. 7). These results show that the internal structure of the nanoparticle influences the thermophoretic forces. Additional simulations of the Janus-1 model using a different mass distribution, $M_a=1645,\ M_b=296$ (see Janus-1$_2$ data in Fig. 7-top) further shows that the internal mass distribution of the particle influences significantly the Soret coefficient.

In addition to thermophoretic drift, thermal gradients induce the alignment of nanoparticles with heterogeneous compositions, such as JNPs [14, 21–23]. The orientation of our Janus nanoparticles changes sign upon increasing the mass ratio, at $M_{a}/M_{b} \sim 1$, with the particles showing significant orientation at high mass ratio, $M_{a}/M_{b}>4$. The lowest mass ratio $M_{a}/M_{b}=0.3$ corresponds to $m_a=m_b$, *i.e.* both shell and core atoms have the same mass, and therefore there is no mass asymmetry in the colloid. In this case, the alignment of the particle is driven by the differences in the solvent-shell and solvent–core interaction strength. Because the shell part (“a”) interacts more strongly with the solvent (leading to a stronger solvation layer, see Fig. 6), the particle orients with “a” towards the cold region, resulting in $\langle \cos \theta \rangle > 0$, as shown in Fig. 7. The preference of the strongest interacting side for the cold region is analogous to what is observed in liquid binary mixtures, where the stronger interacting particles migrate towards the cold region [31]. However, as noted previously in Ref. [22] the mass anisotropy induces an additional torque on Janus particles, leading ultimately to a reversal of the orientation at large enough mass ratios, $M_{a}/M_{b}$.

The orientation of JNPs with high mass anisotropy emerges from the coupling of the mass anisotropy with the heat flux, leading to a thermophoretic torque. The negative sign for the orientation, $\langle \cos \theta \rangle < 0$ observed at $M_{a}/M_{b} > 1$, indicates that the shell part (“a” in the snapshot of Fig. 1-bottom), *i.e.*, the heavier part of the Janus particle, points towards the hot region. This behaviour agrees with that observed in Janus colloids modelled with atomistic and mesoscopic forcefields (see Ref. [22]). The Janus-1 nanoparticle investigated here (see Fig. 1-bottom and data in Fig. 7) does also show orientation, again with the heavier part pointing towards the hot region, $\langle \cos \theta \rangle < 0$, but the orientation is weaker than that of the Janus-2 nanoparticle (see also results for the Janus-1$_2$ case in the same Figure). These results show that the internal composition of the particles matter. To test the impact of internal composition, we performed additional simulations using homogeneous nanoparticles (“Homog.”), namely, removing the mass and interaction anisotropy. We set the total mass of the nanoparticle to 1047, corresponding to the “same mass” particles shown in Fig. 7. The Soret coefficient of the “Homog.” is significantly lower than those obtained with mass anisotropy, highlighting the importance of the internal mass distribution of the nanoparticles in determining the thermophoretic force.

We introduced in a previous work a theory to describe the orientation of Janus colloids featuring mass anisotropy [22]. We showed that the orientation varies with the mass ratio following the Langevin function, $\mathcal {L(\kappa )}$,

4 $$\begin{aligned} \langle \cos \theta \rangle = {\mathcal {L}}(\kappa ) = \coth (\kappa ) - \frac{1}{\kappa } \end{aligned}$$

where $\kappa$ is a parameter that determines the strength of the orientation,

5 $$\begin{aligned} \kappa = -S_T \mu \nabla T \end{aligned}$$

through $\mu$, which is the mass dipole of magnitude, $\mu = | \mathbf{r}_\mathrm{com} - \mathbf{r}_\mathrm{cog} |$, defined by the distance between the center of mass (com) and the center of geometry (cog) of the JNP. The mass dipole depends on the internal geometry of the particle. We show in Fig. 5 in the SI the dependence of the mass dipole with the mass ratio, $M_{a}/M_{b}$. For the same mass ratio, the Janus-2 structure features stronger mass dipoles than the Janus-1 one. This means that for the same total mass, the orientation will be enhanced when the heavy part of the Janus particles is distributed in a thin coating layer. For the specific case of Janus-1 particles, the stronger orientation of the Janus-1$_2$ result shown in Fig. 7 can be understood by comparing the corresponding mass dipoles. For Janus-1$_2$ the mass dipole for $M_a/M_b=1645/296=5.56$ is $\mu =1.54$, higher than $\mu =$1.10 for the Janus-1 model and lower than $\mu =1.96$ for the Janus-2 model. This explains why the orientation obtained for Janus-1$_2$ is between that of the Janus-1 and Janus-2 models.

Equation (4) indicates that the thermal orientation follows a single master curve when represented against $\kappa$. The parameter $\kappa$ in Eq. (5) does not depend explicitly on the interaction strength. However, we showed in Fig. 7 that anisotropic interactions do induce orientation in the particles at zero mass dipole $\mu$. For the systems investigated here, the orientation does not strongly depend on the interaction strength ratio (see Figure 6 in the SI for mass ratios 1.6 and 6.42). Hence, we expect that the mass anisotropy will largely dominate the JNPs orientation. This idea can be tested by checking the degree of orientation of a Janus-2 nanoparticle with $\mu =0$ corresponding to $M_{a}/M_{b} =0.32$. For a large interaction strength ratio, $\varepsilon _{a}/\varepsilon _{b}=10/1$, the orientation is much smaller ($\langle \cos \theta \rangle / \nabla T \sim 5$) in magnitude than the one obtained at high mass ratios (up to $\langle \cos \theta \rangle / \nabla T \sim -15$ for $M_a/M_b = 8$). This result supports the idea that the mass ratio has a larger impact than the interaction strength in driving the orientation of the JNPs with large $M_a/M_b$. Hence, at large $\kappa$ (large mass ratios), we expect the data will follow the functionality predicted by Eq. (4).

**Fig. 8:**
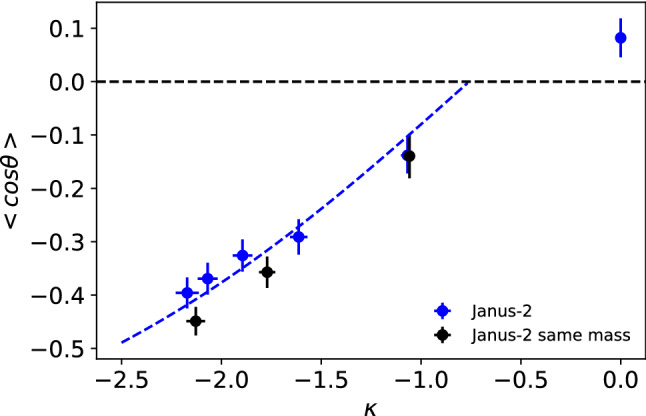
Dependence of the orientation of the Janus-2 nanoparticle with the orientation strength parameter, $\kappa$. The line shows the fitting of Eq. (6) to the “Janus-2” data using the fitting parameter $\kappa _0=-0.759$, and excluding the point at $\kappa =0$ in the fitting.

The functional form ${\mathcal {L}}(\kappa )$ follows from considering the change induced by the thermal gradient on the orientational probability distribution of the nanoparticle, and its corresponding rotational free energy. To model our simulation data, we have used the equation,

6 $$\begin{aligned} \langle \cos \theta \rangle = \coth (\kappa -\kappa _0) - \frac{1}{\kappa -\kappa _0}, \end{aligned}$$

where $\kappa _0$ is a constant that takes into account that $<\cos \theta>$ is not zero at $\mu =0$ due to the contrast in the solvent–nanoparticle interactions. To calculate $\kappa$, we used the data in Fig. 7, the mass dipoles (see Figure 5 in the Supplementary Information) and the average solvent thermal gradient at the nanoparticle centre of mass. The modified Langevin function (Eq. (6)) fits reasonably well to our simulation data for the Janus-2 nanoparticles (see Fig. 8). There are some deviations that might be connected to contributions associated to the interaction ratio, $\varepsilon _a \ne \varepsilon _b$, and the fact that the particles explore slightly different temperature regions due to the orientation around the center of mass.

The main conclusion from the analysis presented above is that the thermal gradients induce orientation in Janus-2 and Janus-1 nanoparticles. For the same mass ratio, the orientation is stronger in the Janus-2 case due to the larger mass dipole. The change in the orientation influences the preference of a specific particle region for the hot and cold areas. The higher affinity of the heavier side for the hot region leads to distinctively different Soret coefficients, as observed in simulations of Janus-2 and Janus-1 nanoparticles, which feature notably different orientations in the thermal field (cf. Janus-2 and Janus-1 results in Fig. 7). The Soret coefficient increases with the particle orientation for particles featuring similar mass.

### Dependence of the Soret coefficient and thermal orientation with temperature

We have shown that for a given thermal gradient, the orientation of the Janus nanoparticle increases with the mass dipole, $\mu$, and the Soret coefficient. We have explored this relationship further by performing additional simulations targeting high and low fluid densities and different temperatures, which result in different solvation structures (see Fig. 6).

**Fig. 9:**
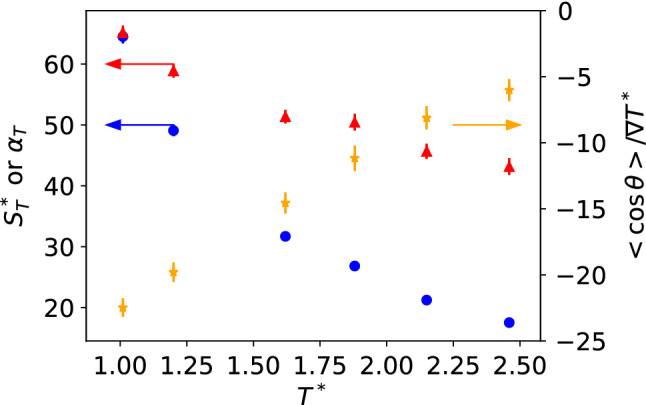
Soret coefficient as a function of temperature. The Janus particle is immersed in a high density liquid or fluid (see stars in Fig. 4). The arrows indicate the y-axis used to represent each data set. Blue-circles, read-triangles and orange stars represent $S_T$, $\alpha _T = S_T^* T^*$ and $\langle \cos \theta \rangle / \nabla T^*$, respectively. All the data correspond to Janus-2 nanoparticles with masses, $m_a=20$, $m_b=1$, $\varepsilon _a=\varepsilon _b=1$.

We discuss in the following simulations of Janus-2 nanoparticles with $m_a=20, m_b=1$ and $\varepsilon _a=\varepsilon _b$. At high solvent density, the Soret coefficient decreases with increasing temperature (see Fig. 9). This dependence is similar to that expected for an ideal system $S_{T, id} = \frac{1}{T}$. However, the JNP is non-ideal as shown by the magnitude of the Soret coefficient and the temperature dependence of the thermal diffusion factor, $\alpha _T= S_T T$, which is not constant (see Fig. 9). Since $\mu$ is constant for all these nanoparticles, and the temperature gradient changes little across different temperatures ($\nabla T^* \sim 0.026{-}0.027$), the weaker orientation at higher temperature must be driven (according to Eqs. (4) and (5)) by the changes in the Soret coefficient.

The direct connection between the Soret coefficient and the thermal orientation suggests that the latter can be enhanced significantly at thermodynamic conditions that lead to a sizeable thermophoretic response. The Soret coefficient is usually small in binary mixtures and solutions, $\sim$10$^{-3}$–10$^{-2}$ K$^{-1}$ and  1 K$^{-1}$ for polymer mixtures. The Soret coefficient of the JNPs investigated here varies between 0.1–0.6 K$^{-1}$ (using $\varepsilon _\mathrm{solvent}/k_B=119$ K as a conversion factor). It has been demonstrated that the Soret coefficient of fluid mixtures increases by several orders of magnitude upon approaching the critical region [3, 32–36]. Approaching the critical temperature from above at constant density, the response functions such as the isobaric thermal expansion increase significantly. Brenner established that the thermophoretic response is proportional to the isobaric thermal expansion coefficient of the solvent [9]. A connection between thermophoresis and the thermal expansion has also been established in reference [37] for dilute solutions. We investigate below the correlation between the Soret coefficient and the isobaric thermal expansion as a function of temperature.

We expect that an enhancement in the Soret coefficient of the JNP will lead to an increase in the thermal orientation, $\left| \langle \cos \theta \rangle \right| \rightarrow 1$, upon approaching the critical point of the Lennard-Jones solvent investigated here. In support of this idea, we note that thermal polarization of polar fluids such as water features an enhancement near the critical point [38]. Furthermore, a relationship has been derived connecting the thermal polarization to the isobaric thermal expansion water [39, 40].

**Fig. 10:**
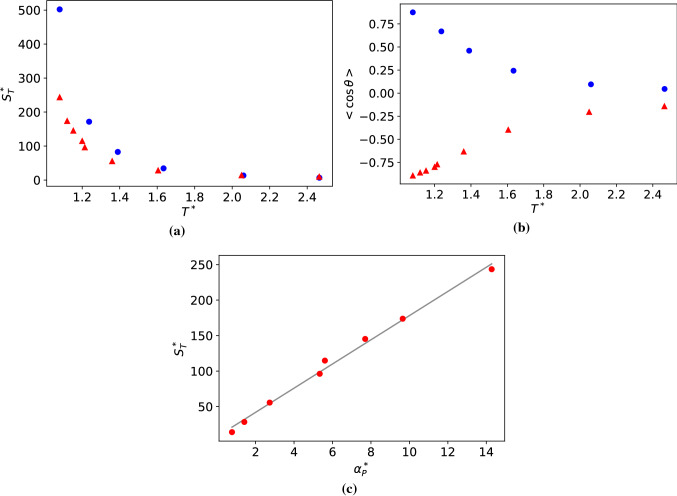
**a** Dependence of the Soret coefficient with the average temperature of the fluid at the colloid position for systems approaching the critical point. **b** Orientation of the JNP as a function of temperature. Blue circles represent data for $m_{a}=m_{b}=1$ and $\varepsilon _{a}=10, \varepsilon _{b}=1$. Red triangles represent $m_{a}=20, m_{b}=1$, and $\varepsilon _{a}=\varepsilon _{b}=1$. **c** Dependence of the Soret coefficient of the Janus-2 nanoparticle $m_{a}=20, m_{b}=1$, $\varepsilon _{a}=\varepsilon _{b}=1$, with the isobaric thermal expansion of the solvent for systems approaching the critical point of the solvent. The grey line represents a linear fitting to simulation data.

We investigated the dependence of the Soret coefficient and the thermal orientation with temperature, targeting thermodynamic states with densities and temperatures in the vicinity of the critical point (see Fig. 4). All the results presented here were obtained with the Janus-2 nanoparticles. We considered two systems: (i) different masses for the core (b) and shell (a), $m_{a} =20$, $m_{b}=1$, and same interaction strength, $\varepsilon _{a}=\varepsilon _{b}=1$, and (ii) Janus particles with the same mass ($m_{a} =m_{b}=1$) and different interaction strengths, $\varepsilon _{a}=10$, $\varepsilon _{b}=1$. We show in Fig. 10a the Soret coefficients as a function of temperature. It is evident that the Soret coefficient increases by 1-2 orders of magnitude for both systems (i) and (ii), upon approaching the critical temperature, $T^*=1.073$, of the solvent. The enhancement of the thermophoresis is accompanied by a significant increase in particle orientation. Close to the critical point, the thermal orientation approaches its limiting value $\left| \langle \cos \theta \rangle \right| \rightarrow 1$ (see Fig. 10b), indicating the particle aligns fully in the direction (or opposite direction) of the thermal gradient. As noted above (following our definition of particle orientation in Fig. 1) particles of types (i) and (ii) orient in opposite directions. For (i), the shell part of the Janus particle orients towards the hot region, and for case (ii), the stronger shell–solvent interaction orients towards the cold region. This behaviour is clearly illustrated in Fig. 10b, with $\langle cos \theta \rangle < 0$ and $\langle cos \theta \rangle > 0$ corresponding to cases (i) and (ii), respectively.

Figure 10c shows the dependence of the Soret coefficient with the isobaric thermal expansion of the solvent. The latter was obtained at different temperatures along the critical isochore, $\rho ^*=0.323$ by differentiating the equation of state of the solvent, $\alpha ^*_P = - (\partial \ln \rho ^*/\partial T^*)_P$ (see Fig. 4). The thermal expansion features an enhancement as the temperature approaches the critical point. We find that the Soret coefficient increases linearly with the isobaric thermal expansion. Microscopically, a larger thermal expansion translates into a stronger density dependence on temperature. For a given thermal gradient, at thermodynamic conditions near the critical point, the Janus nanoparticles will experience larger local density changes induced by the local temperature changes around the nanoparticle (see also Figure 6 for information on the solvation structure). This larger change in density leads to a stronger driving force, and therefore larger Soret coefficient, for the thermophobic nanoparticles studied here, which move towards the higher/lower density/temperature region.

**Fig. 11:**
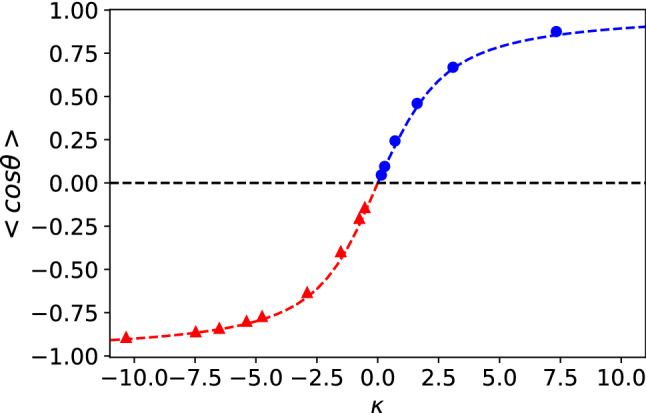
Thermal orientation *vs* the orientation strength, $\kappa$. Blue circles represent $m_{a}=m_{b}=1$ and $\varepsilon _{a}=10, \varepsilon _{b}=1$. Red triangles represent $m_{a}=20, m_{b}=1$, $\varepsilon _{a}=\varepsilon _{b}=1$. The red-dashed line represents the theoretical prediction from Eqs. (4) and (5) and the blue dashed line the prediction using Eq. (4) and $\kappa = -S_T \mu _{\varepsilon } \nabla T$, with $\mu _{\varepsilon }=0.938$, being an adjustable parameter. All the data was obtained with the Janus-2 nanoparticles. The error in the data is of the same order as the symbol size

The overall dependence of the thermal orientation upon approaching the critical point can be understood using Eqs. (4) and (5). For particles with the same interaction core-solvent and shell-solvent ($\varepsilon _a=\varepsilon _b=1$) and different mass ($m_a = 20$, $m_b=1$), the orientation is determined by $\kappa = -S_T \mu \nabla T$ and for same mass dipole and similar $\nabla T$, by $S_T$. The agreement between the simulated orientation and the theory (Eqs. (4) and (5)) is excellent (see Fig. 11). Again, we find that the orientation can be described using the Soret coefficient of heterogeneous particles and the mass dipole.

For particles with the same mass ($m_a=m_b=1$) and different interaction strengths ($\varepsilon _a=10, \varepsilon _b=1$) we cannot use the mass dipole, $\mu$, to define $\kappa$, since $\mu =0$ by definition. Hence, we have modelled our data using Eq. (4) and for the orientation strength, $\kappa = -S_T \mu _{\varepsilon } \nabla T$, where $\mu _{\varepsilon }$ should be a constant for different temperatures, since the interaction strength parameter between the nanoparticle and the solvent does not change with temperature. We show in Fig. 11 that Eq. (4) with the fitting parameter $\mu _{\varepsilon }$ reproduces accurately the orientations of the JNP for $\langle \cos \theta \rangle > 0$.

## Finite size effects

We address in this section the impact of finite size effects on the physical behaviour discussed above. Specifically, we want to know whether the simulation box size influences the dependence of the Soret coefficient and orientation with Janus geometry (Janus-1 *vs* Janus-2), and the Soret/thermal orientation enhancement near the critical point.

We have shown that finite-size effects influence the thermal orientation of dumbbells and trimmers simulated with stochastic rotation dynamics [41], while the impact of the thermal orientation in atomistic simulations of nanorods was negligible. Previous studies using hydrodynamic simulations showed that fixing the position of a colloid in a fluid, as we do here, results in a thermal flow field that influences the magnitude of the Soret coefficient [42, 43]. The boundary conditions affect the velocity field around the nanoparticle, “squeezing” the flow field and imposing a zero velocity boundary condition at the hot and cold thermostats. We show in Figure 7-SI an example of such velocity field for our system. These results were obtained using NEMD simulations of homogeneous nanoparticles with the small simulation boxes ($L^*/R^*=5.08$). The resulting velocity profile is similar to that found using hydrodynamic simulations [43], with a velocity field tangential to the nanoparticle surface, in our case from the cold to the hot region.

**Fig. 12:**
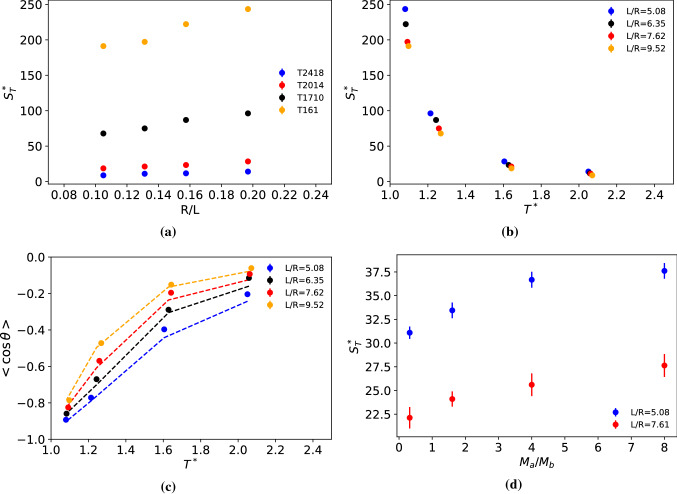
**a** Dependence of the Soret coefficient with box length, *L* for different temperatures approaching the critical point. *L* is the distance between the cold and hot thermostats. Dependence of **b** the Soret coefficient and **c** thermal orientation with temperature and system size. All the results in panels (a-c) were obtained with the Janus-2 nanoparticle $m_a=20$, $m_b=1$, $\varepsilon _a=1$, $\varepsilon _b=1$. The dashed lines in panel **c** represent the results obtained with Eqs. 4–5 using the simulated $S_T$, $\nabla T$ and the mass dipole $\mu =2.01$ (see Fig. 5 in the Supplementary information). **d** Soret coefficient as a function of the mass ratio of the Janus nanoparticle and system size. All the data in panel **d** were obtained with Janus-2 nanoparticles with the “same total mass”. The results for $M_a/M_b=0.32$ correspond to the “Homog.” nanoparticle (see caption Fig. 7).

The Soret coefficient of the Janus nanoparticles investigated here decreases with increasing box size, and the decrease is larger for higher Soret coefficients (see Fig. 12a). We note that we performed the simulation keeping the same temperatures for the hot and cold thermostats; hence the thermal gradient decreases with increasing box length in the order: $\nabla T^* = 0.0247\pm 0.003$ for L/R $=$ 5.08, $\nabla T^* = 0.0191 \pm 0.002$ for L/R $=$ 6.35, $\nabla T^* = 0.0158 \pm 0.002$ for L/R $=$ 7.62 and $\nabla T^*$=$0.0128 \pm 0.001$ for L/R $=$ 9.52, where the error bars indicate the variation in $\nabla T^*$ for systems at different temperatures and the same box size. For thermal gradients listed above, the thermophoretic force features a linear response (see Fig. 3), and hence the magnitude of the gradient does not influence the computed Soret coefficient.

The Soret coefficients feature a significant enhancement near the critical point irrespective of system size (see Fig. 12b), supporting the results presented in (Fig. 10). The thermal orientation increases significantly near the critical point, with the Janus nanoparticle adopting almost full orientation $< \cos \theta >= -1$ at the lowest temperature considered $T^*\sim 1.1$. These results support the existence of a strong thermal orientation effect near a critical point. The decrease of the orientation with increasing system size can be understood using Eqs. (4) and (5), and the changes in $S_T$ and $\nabla T$ for the different systems studied (see dashed lines in Fig. 12c).

We extended our finite-size analysis to the high-density thermodynamic states discussed in Sect. 3.3, focusing on the “same mass” and “homog.” nanoparticles. Following the behaviour reported for low densities, we also find a dependence of the Soret coefficient with increasing system size, and the Soret coefficient becomes smaller (see Fig. 8-SI). The data for larger boxes reproduce the behaviour reported in Fig. 7, namely, nanoparticles with the same total mass but different mass dipoles feature different Soret coefficients. Hence, the simulations with different system sizes support the generality of this physical effect.

## Conclusions

We have investigated the thermophoretic response and thermal orientation of Janus nanoparticles using non-equilibrium molecular dynamics simulations of coarse-grained models. Our simulations show that the thermal orientation is very sensitive to the internal mass distribution of the JNP and, therefore, the internal nanoparticle structure. Structures with the heavy component located in a thin layer at the surface of the JNP result in larger mass dipoles and, therefore, stronger orientation in a thermal field. The orientation of the nanoparticle influences the Soret coefficient. Nanoparticles with the same mass, but different mass distributions, feature different thermophoretic forces. Our results indicate that theoretical approaches aiming at describing the thermophoretic response of colloids in solution must include the effect of the internal mass inhomogeneities, *i.e. the mass dipole*, as a variable. The requirement to include such effects is essential in JNPs, as these particles often feature high mass anisotropy. We conclude that mass anisotropy effects will be prevalent in Janus particles.

The Soret coefficient of Janus particles increases significantly near the solvent critical point, mimicking the behaviour predicted theoretically and observed in experiments of binary mixtures. We found a linear correlation between the Soret coefficient and isobaric thermal expansion coefficient at near critical density conditions. The thermal orientation features significant enhancement too, with the nanoparticles, almost fully aligned with the heat flux at near critical conditions. Since the enhancement is a macroscopic phenomenon, we expect the effect predicted here can be observed in relevant experimental conditions using small thermal gradients.

The specific values of the Soret coefficient and thermal orientation vary with system size. This effect is associated with the dependence of the solvent flow around the fixed nanoparticle, on the distance between the hot and cold boundaries in the simulation box. However, the critical enhancement of the Soret coefficient and the dependence of the thermophoretic response of nanoparticles with different mass anisotropy is reproduced for all the system sizes investigated in this work, supporting the generality of these phenomena.

We have shown that the overall phenomenology of the JNPs thermal orientation in dense fluids and near-critical conditions can be modelled with the Langevin function, $\mathcal {L(\kappa )}$, where $\kappa$ quantifies the orientation strength via $\kappa = -S_T \nabla T \mu$. For a JNP featuring mass asymmetry only (and therefore mass dipole $\mu >0$), the orientation can be predicted directly from a knowledge of the Soret coefficient of the whole nanoparticle. The Langevin function can be extended to model particles with interaction asymmetry, by using a fitting parameter, $\mu _{\varepsilon }$ that accounts for that asymmetry.

We have focused here on a single nanoparticle/solvent size ratio. However, we anticipate that the physical behaviour predicted here should be observed in the large colloid-solvent size regime. We have examined the correspondence between atomistic and mesoscopic regimes before using hydrodynamic simulations (see Ref. [22]). It would be interesting to investigate the impact of the nanoparticle size on the thermophoretic force and nanoparticle thermal orientation, particularly at near-critical conditions using other mesoscopic methods. Furthermore, it will be interesting to establish a correlation between the behaviour reported here and the effective thermal conductivity of the nanoparticles. We note that the definition of the nanoparticle thermal conductivity using bulk properties might not be straightforward, particularly for the shell coated nanoparticles studied here. Therefore, a thermal conductivity analysis will require careful consideration of the correlation between the nanoparticle thermal transport and the internal structure of the nanoparticles.

Our work highlights the importance of both mass and interaction anisotropy on the orientational response of JNPs under thermal fields. Furthermore, the different thermophoretic forces observed in nanoparticles with the same mass underline the importance of the thermal orientation effect discussed in this work. We foresee that the ability to control the behaviour of these fascinating nanomaterials with thermal fields, particularly by modifying their orientation, will open new opportunities in thermal non-equilibrium research in soft matter.

## Supplementary Information

Below is the link to the electronic supplementary material.

Supplementary file 1 (pdf 842 KB)
