# Supplementary material for: Thermophoresis and thermal orientation of Janus nanoparticles in thermal fields
Source: Eur Phys J E Soft Matter. 2022 Jul 9;45(7):59. doi: 10.1140/epje/s10189-022-00212-3 (PMC9271122; doi:10.1140/epje/s10189-022-00212-3)
Supplement: Supplementary file 1 — (pdf 842 KB) [file 10189_2022_212_MOESM1_ESM.pdf]

Supplementary Information: Thermophoresis and thermal orientation of Janus Nanoparticles in thermal fields

Fernando Bresme, Juan D. Olarte-Plata, Aidan Chapman, Pablo Albella, Calum Green

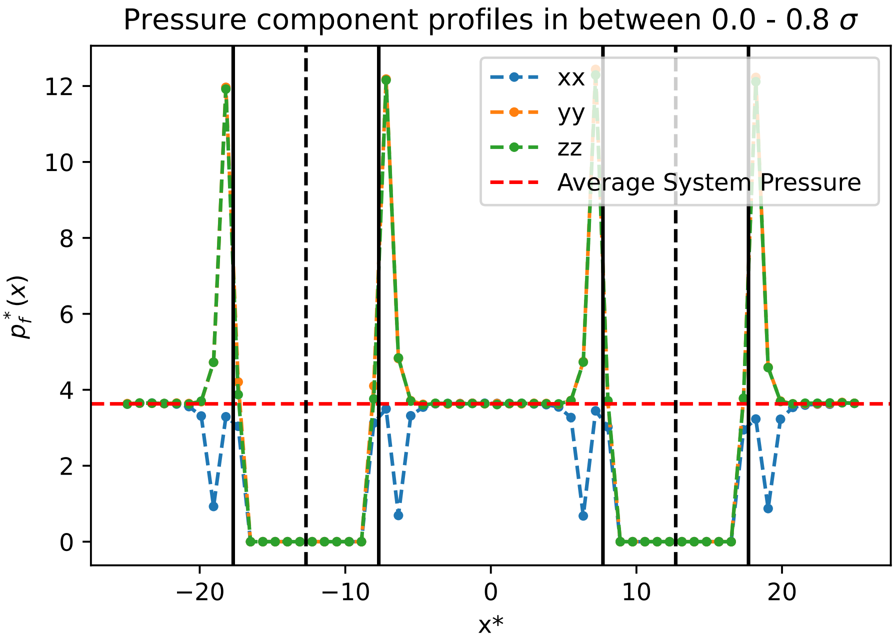

Fig. 1: Pressure profile along the heat flux direction (x) for a cylindrical volume of radius 0.8σ, centered at the centre of mass of a homogeneous nanoparticle with $\varepsilon_a = \varepsilon_b = 10$, $m_a = m_b = 1$ in a fluid of density $\rho^* = 0.8$ and under a thermal gradient obtained with $T^*_{HOT} = 1.8$ and $T^*_{COLD} = 1.2$. The average system pressure was obtained from the average of the diagonal components of the pressure tensor of the whole system.

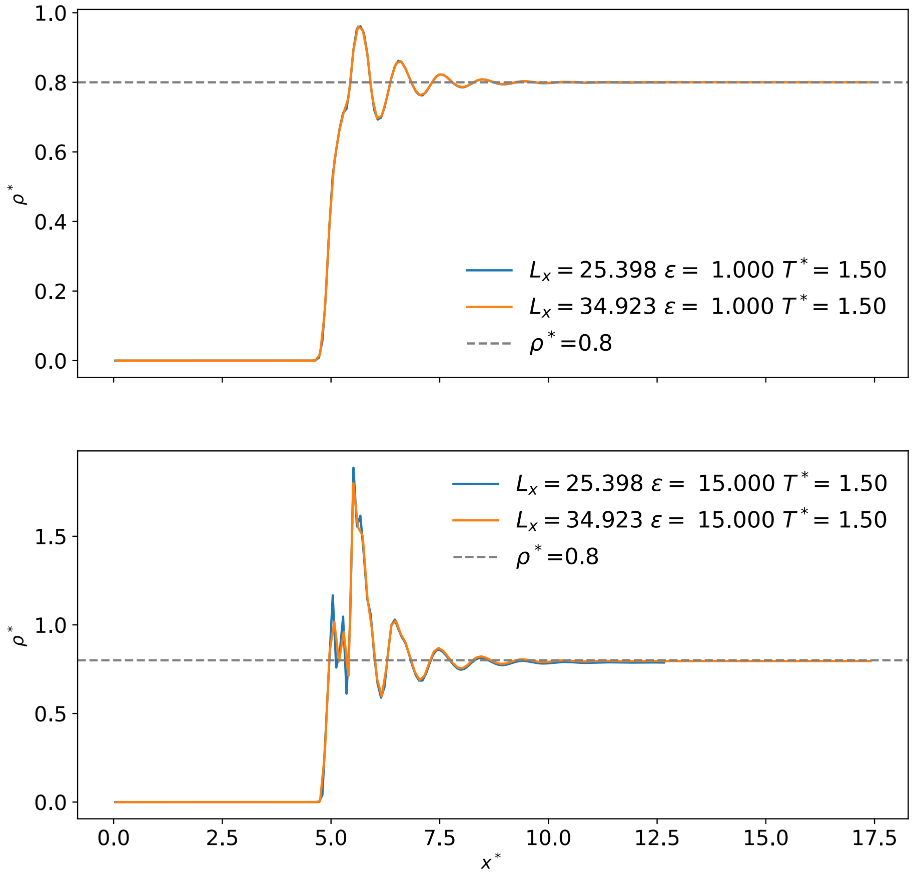

Fig. 2: Radial density profiles of equilibrium simulations performed with simulation boxes of different sizes In both cases $L_y^* = L_z^*$ and $L_x^* = 2L_y^*$. No significant finite-size effects were observed in the radial density profiles around the particles.

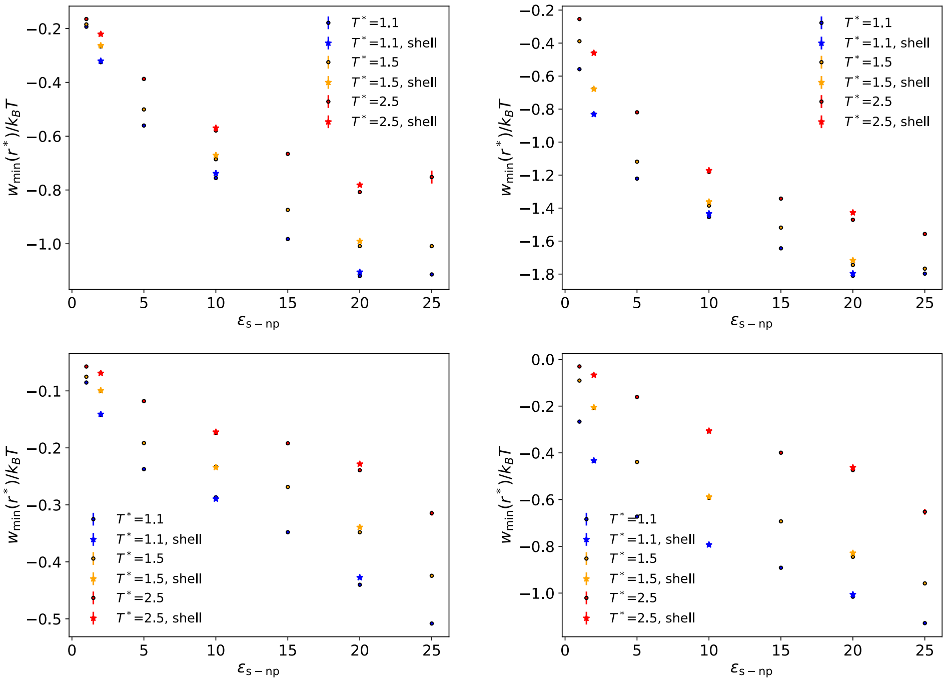

Fig. 3: (Top two panels) First minimum of the PMF for different nanoparticle-solvent interaction strengths, ε_{s-np} and temperatures, for solvent densities of 0.8 (left) and 0.4 (right). (Bottom two panels) Second minimum in the PMF for different nanoparticle interaction strengths, for solvent bulk densities of ρ^*=0.8 (left) and 0.4 (right). The homogeneous nanoparticles are represented with dots in all four plots, whilst the non-Janus core-shell particles are represented with stars.

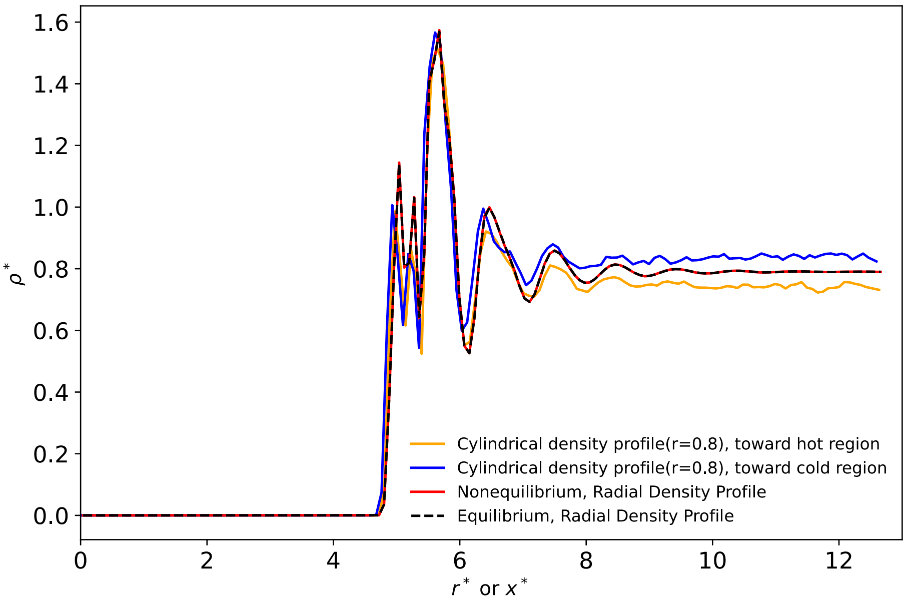

Fig. 4: Non-equilibrium simulations with homogeneous particles $\varepsilon_a = \varepsilon_b = 10$, $m_a = m_b = 1$, $T^*_{HOT} = 1.8$, $T^*_{COLD} = 1.2$ and an average density of $\rho^* = 0.8$. The density profiles, pointing toward the hot thermostat (orange line) and toward the cold (blue line), were obtained using a cylindrical sampling volume of radius 0.8σ with axial distance along the x-axis. These profiles are compared to the radial density profiles (using spherical shells) obtained with equilibrium simulations at the same average density and temperature (dotted black line) and the corresponding radial density profile in the NEMD simulation (red line). The x-axis represents either the radius of the spherical shells (r^*) or the cylinder's axial distance along the x-coordinate (x^*). Note that the cylindrical profiles converge to different densities at long distances from the nanoparticle, reflecting the lower/higher densities of the solvent in the hot/cold regions.

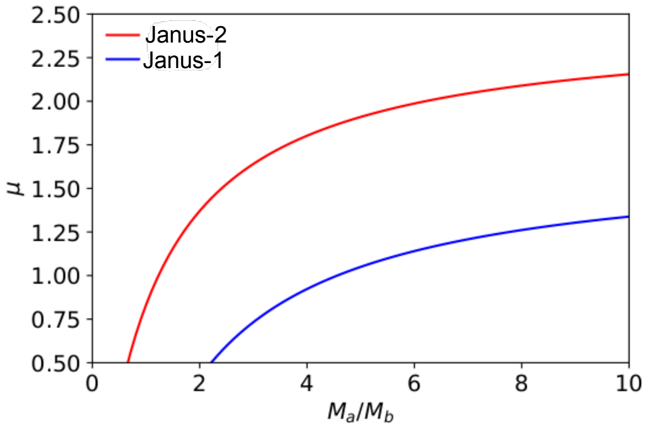

Fig. 5: Mass dipole, μ, vs the ratio of the total masses of the shell(a) and core (b) parts of the JNP.

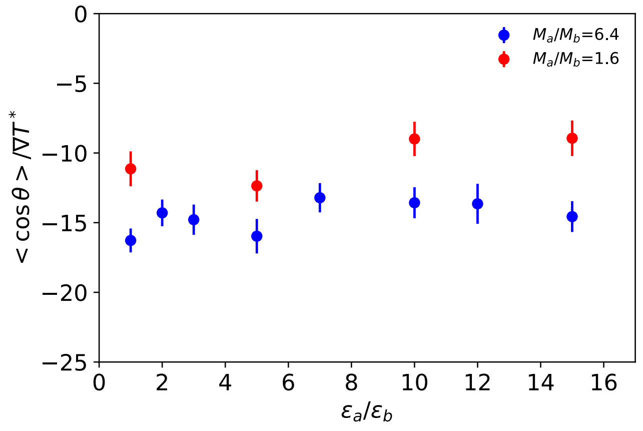

Fig. 6: Dependence of the thermal orientation with the interaction ratio of shell(a) and core(b) parts of the Janus particle. $\varepsilon_b = 1$. $m_a = 20$ and $m_b = 1$ for $M_a/M_b = 6.4$, and $m_a = 5$ and $m_b = 1$ for $M_a/M_b = 1.6$.

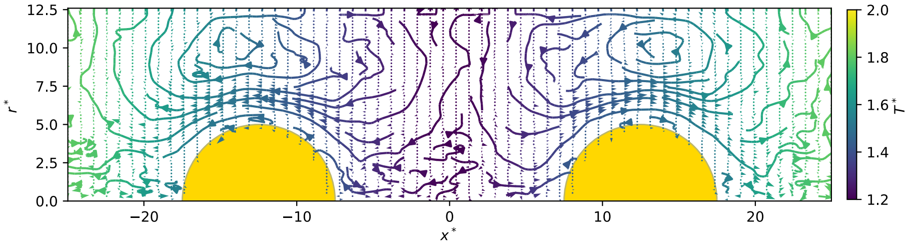

Fig. 7: Velocity field around homogeneous ($\varepsilon_a = \varepsilon_b = 10$, $m_a = m_b = 1$) nanoparticles under a thermal gradient with $T^*_{HOT} = 1.8$, $T^*_{COLD} = 1.2$, and average fluid density of $\rho^* = 0.8$. The arrows indicate the direction of the velocity in x and radial (perpendicular to the heat flux) directions. The radial component of the velocity of a particle i was obtained using $v_{i,r} = (v_{i,y}r_{i,y} + v_{i,z}r_{i,z})/\sqrt{r^2_{i,y} + r^2_{i,z}}$. The local velocities were obtained using cylindrical shells of radial thickness 0.2117σ and thickness 0.8466σ in the x direction, centred about the cylinder axis (x-component). The velocity field was obtained using 29 independent simulation trajectories each with at least 3×10^6 steps. The cold thermostat is located at $x^* = 0$.

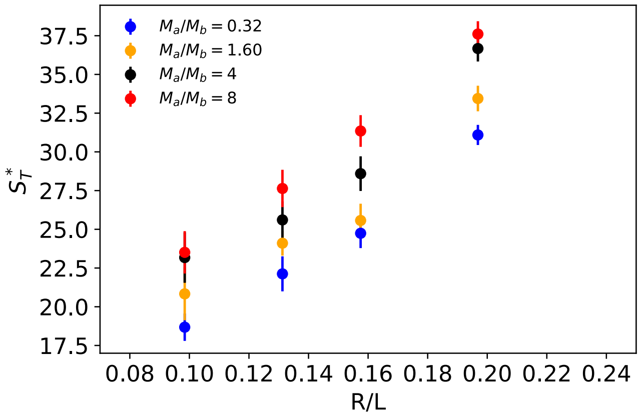

Fig. 8: Dependence of the Soret coefficient with box length for the Janus-2 nanoparticles "same mass" system represented in Figure 7 in the main text. The results for $M_a/M_b = 0.32$ correspond to the "Homog." nanoparticle (see caption Figure 7).
